# Co‐Culture system for studying neuro‐inflammation pathology with iPSC derived cerebral organoid and microglia

**DOI:** 10.1002/alz70855_097165

**Published:** 2025-12-23

**Authors:** Wonyoung Koh, Gwang‐Hyun Park, Yoomin Park, Jinguen Rheey, Jeyoung Ryu

**Affiliations:** ^1^ GradiantBioconvergence, Songpa‐Gu, Seoul, Korea, Republic of (South)

## Abstract

**Background:**

Alzheimer's disease (AD), the most common form of dementia, is pathologically defined by amyloid‐β plaques and hyperphosphorylated tau protein, with growing evidence highlighting neuroinflammation, as a critical contributor to disease. Recent advances in induced pluripotent stem cell (iPSC) technology have enabled the generation of three‐dimensional cerebral organoids that better recapitulate human neurodevelopmental processes than conventional two‐dimensional models. By integrating microglia into these organoids, researchers can more accurately mimic the inflammatory environment of the AD brain and elucidate the interactions between microglia and neurons. This co‐culture platform not only provides insight into how neuroinflammation evolves AD pathology, but also be a useful tool for identifying potential therapeutic target.

**Method:**

Healthy controls and sporadic Alzheimer's disease (AD) patients‐derived iPSC differentiated each line into cerebral organoids and microglia. These were then co‐cultured autologously to maintain donor specificity. To induce a neuroinflammatory phenotype, we treated the co‐cultures with lipopolysaccharide (LPS). We further applied a chemical inhibitor to evaluate its impact on pro‐inflammatory cytokine production in the conditioned media.

**Result:**

LPS‐induced inflammatory responses were only observed when microglia were present in the co‐culture. While some variation was noted among different cell lines, pro‐inflammatory markers, including TNF‐α, IL‐6, and IL‐1β, were markedly elevated upon LPS treatment. Notably, administering the JNK inhibitor SP600125 significantly reduced the levels of these cytokines. Immunostaining confirmed that microglia within the organoids were capable of phagocytosing amyloid‐β, and co‐cultures exhibited differences in amyloid‐β levels compared to organoids without microglia.

**Conclusion:**

Traditional cerebral organoid models have been limited by the difficulty in differentiating microglia or the prolonged periods required for their maturation. Here, we established a more physiologically relevant system by co‐culturing organoids and microglia at a developmentally analogous timepoint, enhancing our ability to mimic in vivo brain processes. Moreover, by employing AD patient‐derived iPSCs, we successfully recapitulated disease‐associated inflammatory phenotypes, highlighting the potential of this platform for both mechanistic studies and drug screening in the context of AD.

